# Molecular Epidemiology of Enterovirus 71 Infection in the Central Region of Taiwan from 2002 to 2012

**DOI:** 10.1371/journal.pone.0083711

**Published:** 2013-12-31

**Authors:** Wen-Hao Wu, Ta-Cheng Kuo, Yu-Ting Lin, Szu-Wei Huang, Hsin-Fu Liu, John Wang, Yi-Ming Arthur Chen

**Affiliations:** 1 Institute of Public Health, National Yang-Ming University, Taipei, Taiwan; 2 Department of Pediatrics, Wan Fang Hospital, Taipei, Taiwan; 3 Center for Infectious Disease and Cancer Research, Kaohsiung Medical University, Kaohsiung, Taiwan; 4 Institute of Microbiology and Immunology, School of Life Sciences, National Yang-Ming University, Taipei, Taiwan; 5 Department of Medical Research, Mackay Memorial Hospital, Taipei, Taiwan; 6 Department of Pathology, Taichung Veterans General Hospital, Taichung, Taiwan; 7 Department of Microbiology and Institute of Medical Research, College of Medicine, Kaohsiung Medical University, Kaohsiung, Taiwan; University of Illinois at Chicago, United States of America

## Abstract

Enterovirus 71 (EV71), a causative agent of hand, foot, and mouth disease can be classified into three genotypes and many subtypes. The objectives of this study were to conduct a molecular epidemiological study of EV71 in the central region of Taiwan from 2002–2012 and to test the hypothesis that whether the alternative appearance of different EV71 subtypes in Taiwan is due to transmission from neighboring countries or from re-emergence of pre-existing local strains. We selected 174 EV71 isolates and used reverse transcription-polymerase chain reaction to amplify their VP1 region for DNA sequencing. Phylogenetic analyses were conducted using Neighbor-Joining, Maximum Likelihood and Bayesian methods. We found that the major subtypes of EV71 in Taiwan were B4 for 2002 epidemic, C4 for 2004–2005 epidemic, B5 for 2008–2009 epidemic, C4 for 2010 epidemic and B5 for 2011–2012 epidemic. Phylogenetic analysis demonstrated that the 2002 and 2008 epidemics were associated with EV71 from Malaysia and Singapore; while both 2010 and 2011–2012 epidemics originated from different regions of mainland China including Shanghai, Henan, Xiamen and Gong-Dong. Furthermore, minor strains have been identified in each epidemic and some of them were correlated with the subsequent outbreaks. Therefore, the EV71 infection in Taiwan may originate from pre-existing minor strains or from other regions in Asia including mainland China. In addition, 101 EV71 isolates were selected for the detection of new recombinant strains using the nucleotide sequences spanning the VP1-2A-2B region. No new recombinant strain was found. Analysis of clinical manifestations showed that patients infected with C4 had significantly higher rates of pharyngeal vesicles or ulcers than patients infected with B5. This is the first study demonstrating that different EV 71 genotypes may have different clinical manifestations and the association of EV71 infections between Taiwan and mainland China.

## Introduction

Enterovirus 71 (EV71), a member of the genus of *Enterovirus* in the *Piconaviridae* family, is a positive-stranded RNA virus with a genome approximately 7,400 bases long [1]. EV71 causes hand-foot-mouth disease (HFMD) and could potentially cause severe neurologic complications resulting in the death of young children [2], [3]. EV71 is classified into three genotypes-A, B and C based on phylogenetic tree analysis using VP1 nucleotide sequences [4]. Genotypes B and C are further divided into subtypes- B1 to B5 and C1 to C5, respectively [4], [5].

EV71 was first isolated in California in 1969 and subsequently, both endemic and epidemic infections were found in different regions of the world [5]. It has been reported that genotype B is predominant in southeastern Asia, including Malaysia, Brunei and Singapore, whereas genotype C is predominant in northeast Asia, including China, Vietnam and Korea [5]. Another important aspect of the molecular epidemiology of EV71 is the existence of recombinant strains. Furthermore, the recombination happens not only between different EV71 subtypes, but also between EV71 and Coxsackie virus A strains [6], [7]. For example, subtype C4 is a recombinant between B2 and C2, while subtype B4 is a recombinant between B3 and B2 [7]. Since the recombination happened frequently in the 2A, 3D and 5′UTR regions [8], we established a RT-PCR method using a panel of primer pairs spanning VP1-2A-2B (Information S1), 5′UTR and 3′UTR regions for the detection of new EV71 recombinant strains in this study.

In Taiwan, EV71 subtypes C2 and B4 caused about 1.5 million infections and resulted in 78 deaths in 1998 [9]. Subsequently, the following EV71 subtypes have appeared: B4 in 1999, C4 in 2004–2005, C5 in 2006–2007, and B5 in 2008 [10]–[12]. Since Taiwan is located in the central region of Asia, it is important to know whether this alternative appearance of different EV71 subtypes is due to transmission from neighboring countries, re-emergence from pre-existing local strains or both. Although HFMD occurred earlier in the central region than other regions in Taiwan (personal communication, Dr. Chuang, Jen-Hsiang, Taiwan Centers for Disease Control), there was only one molecular epidemiological study conducted in this region in 2005 and the number of isolates analyzed in the study was very small [13]. Therefore, we decided to conduct a molecular epidemiological study using 174 EV71 isolates from patients with HFMD in the central region of Taiwan from 2002–2012. In addition, we used phylogenetic tree analysis to elucidate the possible origin of these EV71 subtypes. Finally, clinical data showed that different EV71 subtypes may have distinct clinical manifestations of the HFMD.

## Subjects, Materials and Methods

### Subjects and EV71 Isolates

From January 2002 to March 2012, a total of 218 EV71 isolates were collected from patients with confirmed or suspected diagnosis of HFMD at the Central Region Contract Laboratory (CRCL) of Taiwan CDC. The CRCL is located at the Taichung Veterans General Hospital and responsible for the laboratory diagnosis of enterovirus infection in the central region of Taiwan. To conduct a molecular epidemiological study of EV71, we selected all the specimens collected in different years except 2008 in which we randomly selected 51 of 91 isolates for the analysis. All the personal information of the patients was replaced with different codes before their demographic data and clinical manifestations were analyzed. This study was approved by the institutional reviewing board of Taichung Veterans General Hospital. Since it is an unlinked anonymous database analysis, written consent was not obtained from the participants and it has been approved by the institutional reviewing board.

### Reverse Transcription-Polymerase Chain Reaction (RT-PCR) for EV71 and DNA Sequencing

Viral RNA was extracted from cultured medium using a QIAmp Viral RNA mini Kit (QIAgen, Hilden, Germany). Reverse transcription reaction was performed using a SuperScript II Kit with random hexamers (Invitrogen, Carlsbad, CA, USA). A semi-nested PCR method was developed using degenerative primers which published previously (Information S1) [7]. For molecular epidemiological study, the VP1 gene of EV71 was amplified using primers EV3F and EV3R-2. Subsequently, 101 isolates were selected for PCR analysis using primer pairs covering the remaining genes of the VP1, VP2A and VP2B region (Information S1). Semi-nested PCR was conducted using primers EV3F and EV5R in the first round of PCR and the following primer pairs in the second round PCR for different regions: primers EV3F and EV3R-2 for VP1; primers EV4F-2, EV5R for VP2A-VP2B regions. The PCR products were sequenced using a DNA analyzer (ABI 3730XL, Carlsbad, CA, USA).

### Phylogenetic Analysis

Phylogenetic trees were constructed using the Find Best Model methods in MEGA 5 [14]. Both Neighbor-Joining and Maximum Likelihood methods with Kimura 2-parameter model plus gamma distribution and 1000 bootstrap replicates were performed using MEGA 5 programs. To identify the origin and dissemination of different Taiwanese EV71 strains, we included VP1 sequences from 190 EV71 reference strains for the phylogenetic analysis. The reference sequences were obtained through using the NCBI Basic Local Alignment Search Tool (BLAST) program (http://blast.ncbi.nlm.nih.gov/Blast.cgi) as well as through literature review. Besides, to obtain a stable tree, at least 2 strains from all the EV71 subtypes were included during the construction of the trees. The sequences from Coxsackie virus group A type 16 were used as the out group in the rooted trees. To identify the recombinant events, trees were constructed using Tamura-Nei model plus gamma distribution with 1,000 bootstrap replicates in MEGA 5. A bootstrap value of 70 was used as an indicator of the significance of the clusters.

### Accession numbers of the EV71 sequences

The GenBank accession numbers for the VP1-2A-2B and partial 2C gene of EV71 in Taiwan from 2002 to 2012 were JX991043-JX991143. The GenBank accession numbers for the VP1 gene of EV71 in Taiwan from 2002 to 2012 were JX990970-JX991042.

### Statistical Analysis

Fisher's exact and χ^2^ tests were performed using SAS v8.2. to determine the statistical significance for the comparison of the clinical manifestations between patients with EV71 B5 and C4 infection.

## Results

**Figure 1:**
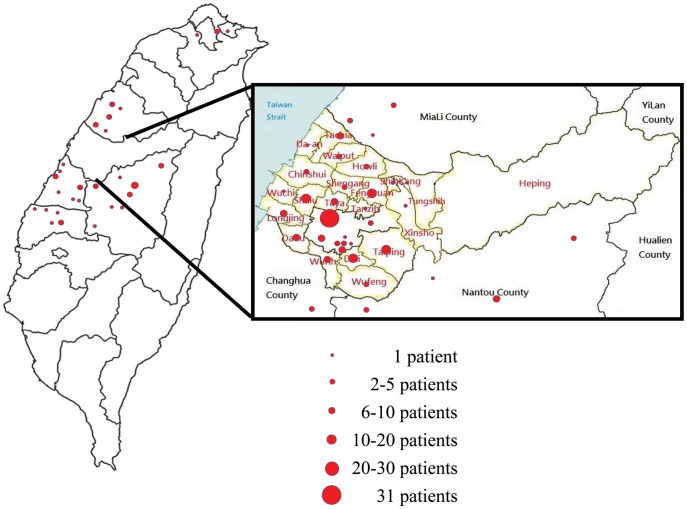
Geographic distribution of EV71-infected patients participated in this study from 2002 to 2012 in Taiwan.

**Figure 2:**
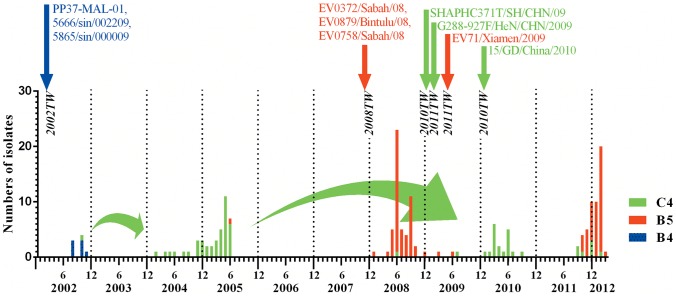
Temporal distribution of different EV71 subtypes in Taiwan from 2002 to 2012 and their possible origins.

As shown in Figure 1, the geographic distribution of the patients with HFMD in this study was mainly in the central region of Taiwan. For molecular epidemiological study, we used all the EV71 isolates collected at the CRCL of Taiwan CDC from 2002-2012 except 2008 since there were 91 cases during that year. We eventually selected 51cases randomly from the outbreak in 2008 for the study. The VP1 gene was amplified using a semi-nested RT-PCR method and the PCR products were sequenced. Subsequently, the EV71 subtypes were determined using phylogenetic tree analysis with both Neighbor-Joining (NJ) and Maximum Likelihood (ML) methods. As shown in Figure 2, in 2002, the main subtype of EV71 infection in the central region of Taiwan was subtype B4 (7/8 [87.5%]), with only 1 subtype C4 isolate (12.5%). In 2004-2005 epidemic, the EV71 major subtype changed to C4 (41/42 [97.6%]), with only 1 subtype B5 isolate (2.4%) found in 2005. In 2008–2009 epidemic, the major subtype of EV71 changed to B5 (52/54 [96.3%]), with only two strains of EV71 subtype C4 (3.7%). In 2010, all the EV71 subtype was C4. Finally, in 2011–2012 epidemic, subtype B5 was predominant (45/52, 86.5%), with sporadic subtype C4 (7/52, 13.5%) infections.

### Clinical epidemiological study of EV71 subtypes in the central region of Taiwan

We compared the clinical diagnosis, symptoms and signs between patients infected with subtypes B5 and C4 and the results showed that patients with subtype B5 had significantly high rate of HFMD diagnosis than that of patients with subtype C4 infection (77.4% vs. 59.7%, *p*<0.05). In addition, patients with subtype C4 had significant higher rates of the following symptoms than patients with subtype B5 infection: pharyngeal vesicles or ulcers (35.5% vs. 9.4%, p = 0.001), rhinorrhea (11.3% vs. 1.9%, p<0.05) and cough (16.1% vs. 3.8%, p<0.05) (Table 1). It indicates that patients infected with C4 may have higher rates of atypical HFMD symptoms.

**Table 1:** Comparison of the diagnosis of hand-foot-mouth disease and symptoms and signs between patients infected with enterovirus 71 subtypes B5 and C4.

	No. (%) of Children			
Diagnosis or symptoms	B5	C4	Total	*p* value
	(N = 53)	(N = 62)	(N = 115)	
Diagnosed with HFMD*				
Yes	41 (77.4)	37 (59.7)	78 (67.8)	<0.05
*Suspected	12 (22.6)	25 (40.3)	37 (32.2)	
Symptoms				
Fever	36 (67.9)	41 (66.1)	77 (67.0)	
Skin rash	19 (35.9)	17 (27.4)	36 (31.3)	
Herpangina	5 (9.4)	22 (35.5)	27 (23.5)	0.001
Sore throat	9 (17.0)	10 (16.1)	19 (16.5)	
Cough	2 (3.8)	10 (16.1)	12 (10.4)	<0.05
Vomit	5 (9.4)	4 (6.5)	9 (7.8)	
Rhinorrhea	1 (1.9)	7 (11.3)	8 (7.0)	<0.05
Paralysis	0	2 (3.2)	2 (1.7)	
Headache	0	2 (3.2)	2 (1.7)	
Prostration	0	2 (3.2)	2 (1.7)	
Influenza-like illness	0	2 (3.2)	2 (1.7)	
Diarrhea	0	1 (1.6)	1 (0.9)	
Myalgia	0	1 (1.6)	1 (0.9)	

### Origin and dissemination of EV71 in the central region of Taiwan

**Figure 3:**
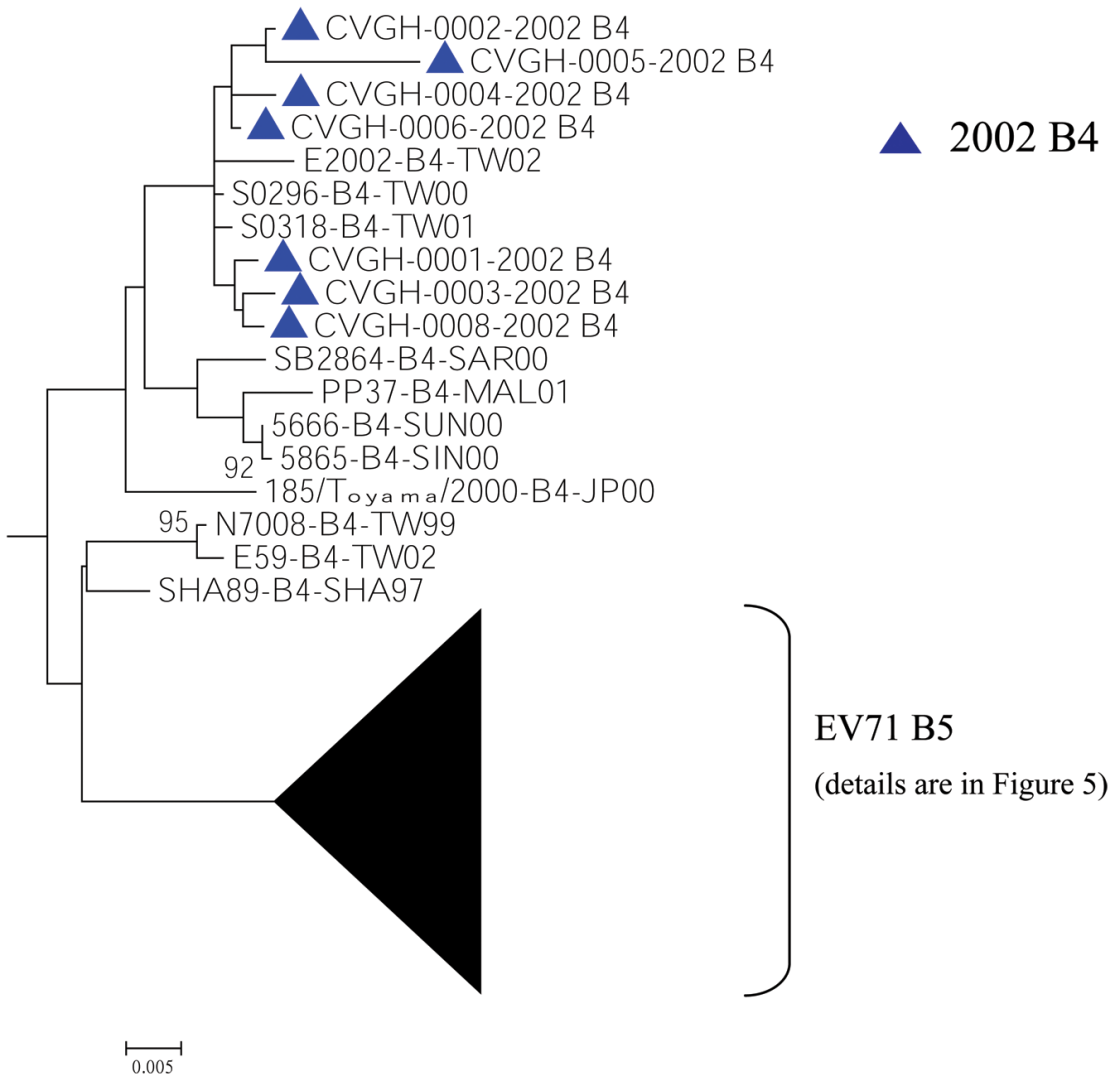
Phylogenetic tree analysis of different Taiwanese and Asian EV71 isolates using Maximum likelihood method with nucleotide sequences from VP1 region (890 nucleotides in length, the subtype B4 clade). Bootstrap analysis was performed using 1000 times.

To elucidate the origin and dissemination pathway of EV71 in the central region of Taiwan, the VP1 sequences of 174 Taiwanese EV71 strains from this study together with 190 reference stains that we obtained from GenBank were used for phylogenetic tree analysis (Information S2). As showed in Figure 3, the EV71 subtype B4 outbreak in 2002 in Taiwan clustered with EV71 strains from Malaysia (PP37) and Singapore (5666 and 5865) which had been isolated in 2000 and 2001. Although the bootstrap value using Maximum Likelihood method was lower than 50, the bootstrap value using Neighbor-Joining method was 73.

**Figure 4:**
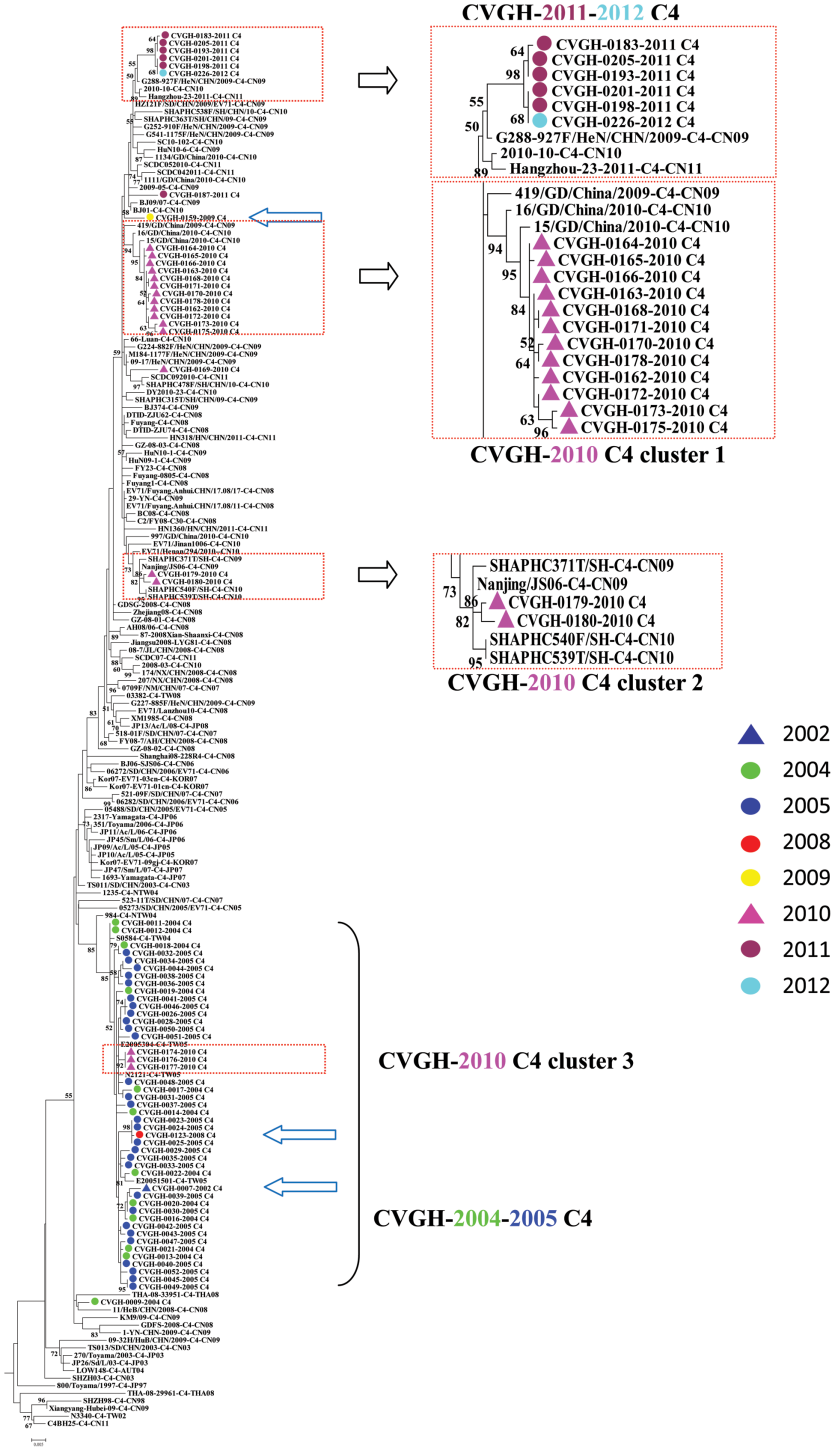
Phylogenetic tree analysis of different Taiwanese and Asian EV71 isolates using Maximum likelihood method with nucleotide sequences from VP1 region (890 nucleotides in length, the subtype C4 clade). Bootstrap analysis was performed using 1000 times. Arrows: minor strains of EV71 found in different epidemics.

Concerning the origin of EV71 subtype C4 strains in Taiwan, all the C4 isolates from 2005 clustered with most Taiwanese strains isolated in 2004 (Figure 4). Therefore, they belonged to the same endemic. It is intriguing to note that the minor strain C4 (CVGH0007-2002) that we identified in 2002 clustered with those C4 strains in 2004–2005. In terms of those 18 subtype C4 strains that we isolated in 2010, they formed the following three clusters with isolates from other countries: cluster 1 consists of 12 (66.7%) CVGH isolates which clustered with one EV71 strain (15/GD/China/2010) reported in 2010 in Guangdong Province, China with a bootstrap value of 95; cluster 2 consists of two (11.1%) CVGH isolates which clustered with one EV71 strain (SHAPHC371T/SH/CHN/09) from Shanghai, China isolated in 2009 with a bootstrap value of 82; the third cluster consists of 3 (16.7%) Taiwanese isolates which clustered with other EV71 strains isolated in 2004 and 2005 with bootstrap value of 92. It indicates that EV71 infections in the central region of Taiwan originate from previously existing strains from either local or foreign countries. Finally, for subtype C4 strains in 2011–2012, they clustered with one EV71 strain (G288-927F/HeN/CHN/2009) from Henan Province, China isolated in 2009 with bootstrap value of 55.

**Figure 5:**
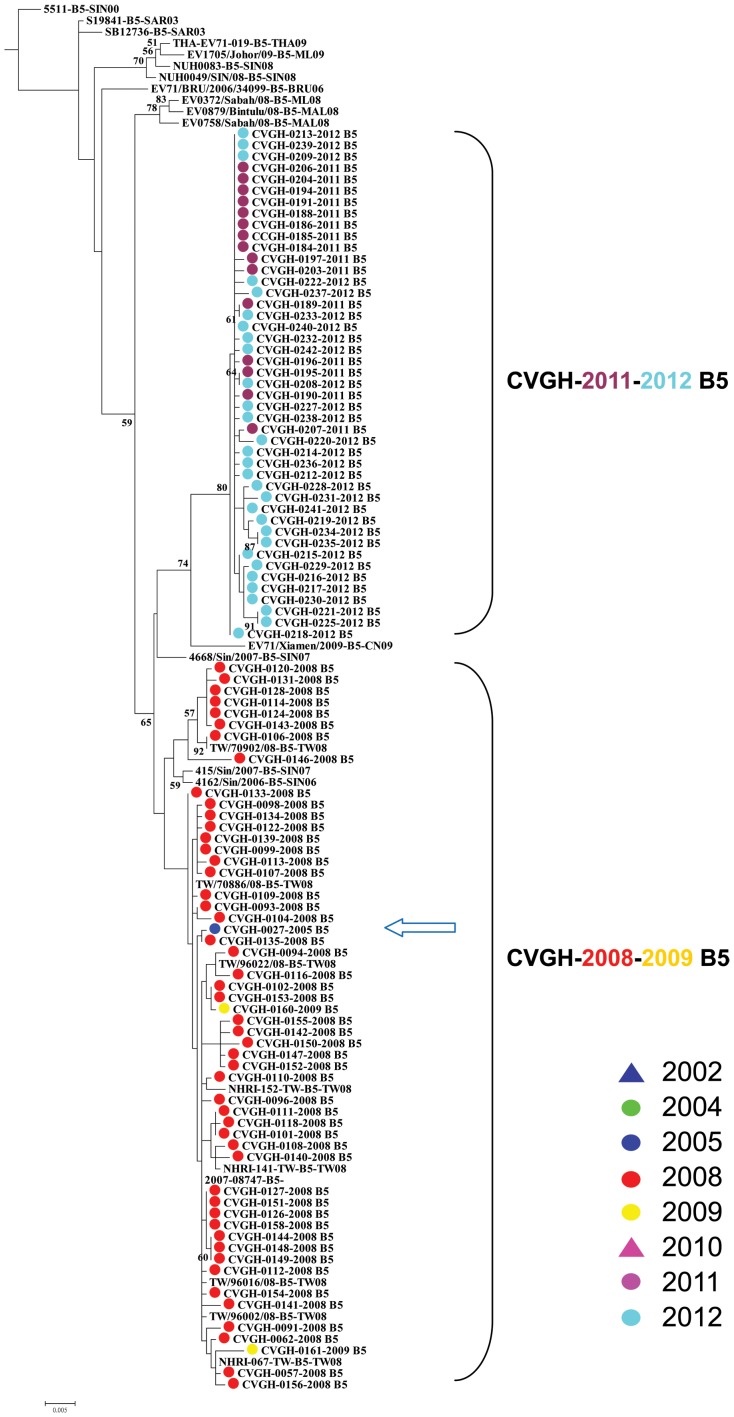
Phylogenetic tree analysis of different Taiwanese and Asian EV71 isolates using Maximum likelihood method with nucleotide sequences from VP1 region (890 nucleotides in length, subtype B5 clade). Bootstrap analysis was performed using 1000 times. Arrows: minor strains of EV71 found in different epidemics.

In terms of the origin of the subtype B5 which responsible for the major EV71 infections in 2008 and in 2011–2012, phylogenetic analysis only showed that the 2011–2012 B5 strains clustered with one EV71 strain (EV71/Xiamen/2009) from Xiamen City, Fu-Chien Province, China in 2009 with bootstrap value of 74 (Figure 5). The results were confirmed using Neighbor-Joining method with bootstrap value of 68.

### Detection of recombinant EV71 strains

**Figure 6:**
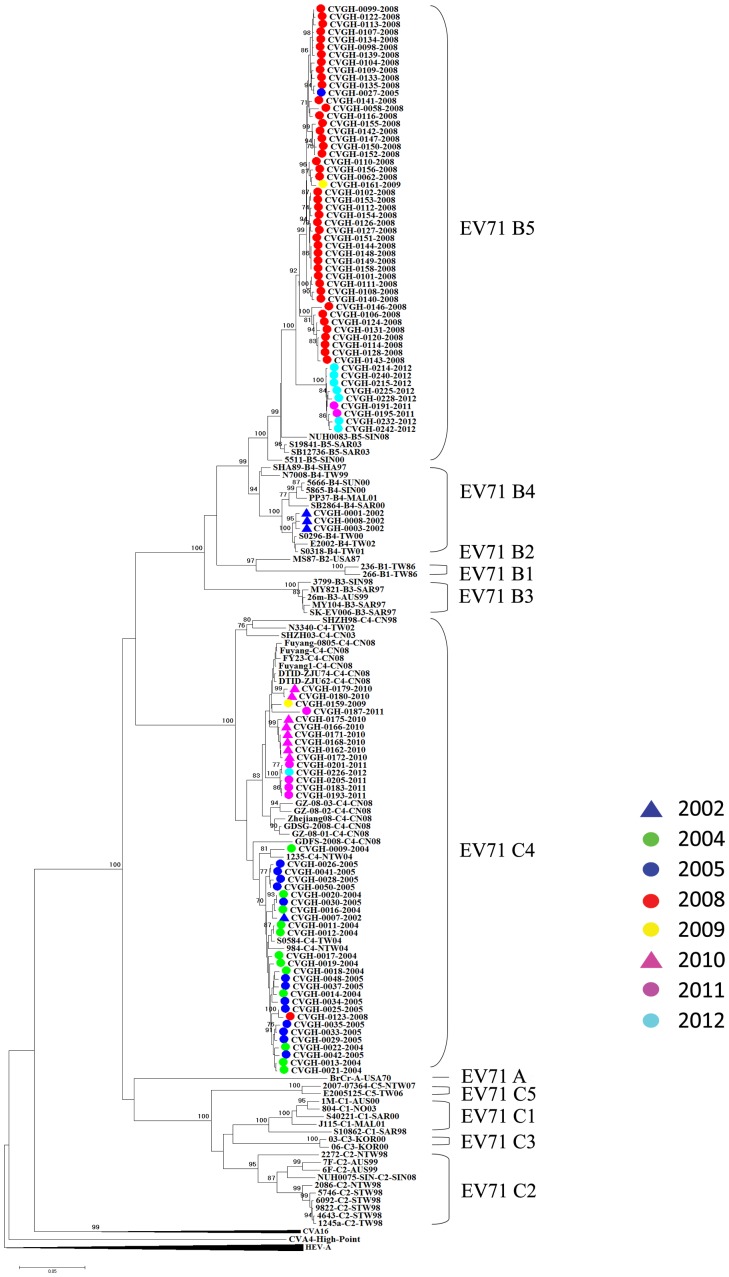
Phylogenetic tree analysis of different Taiwanese EV71 isolates using neighbor joining method with nucleotide sequences from VP1-VP2A-VP2B regions (2281 nucleotides in length). Bootstrap analysis was performed using 1000 times.

**Figure 7:**
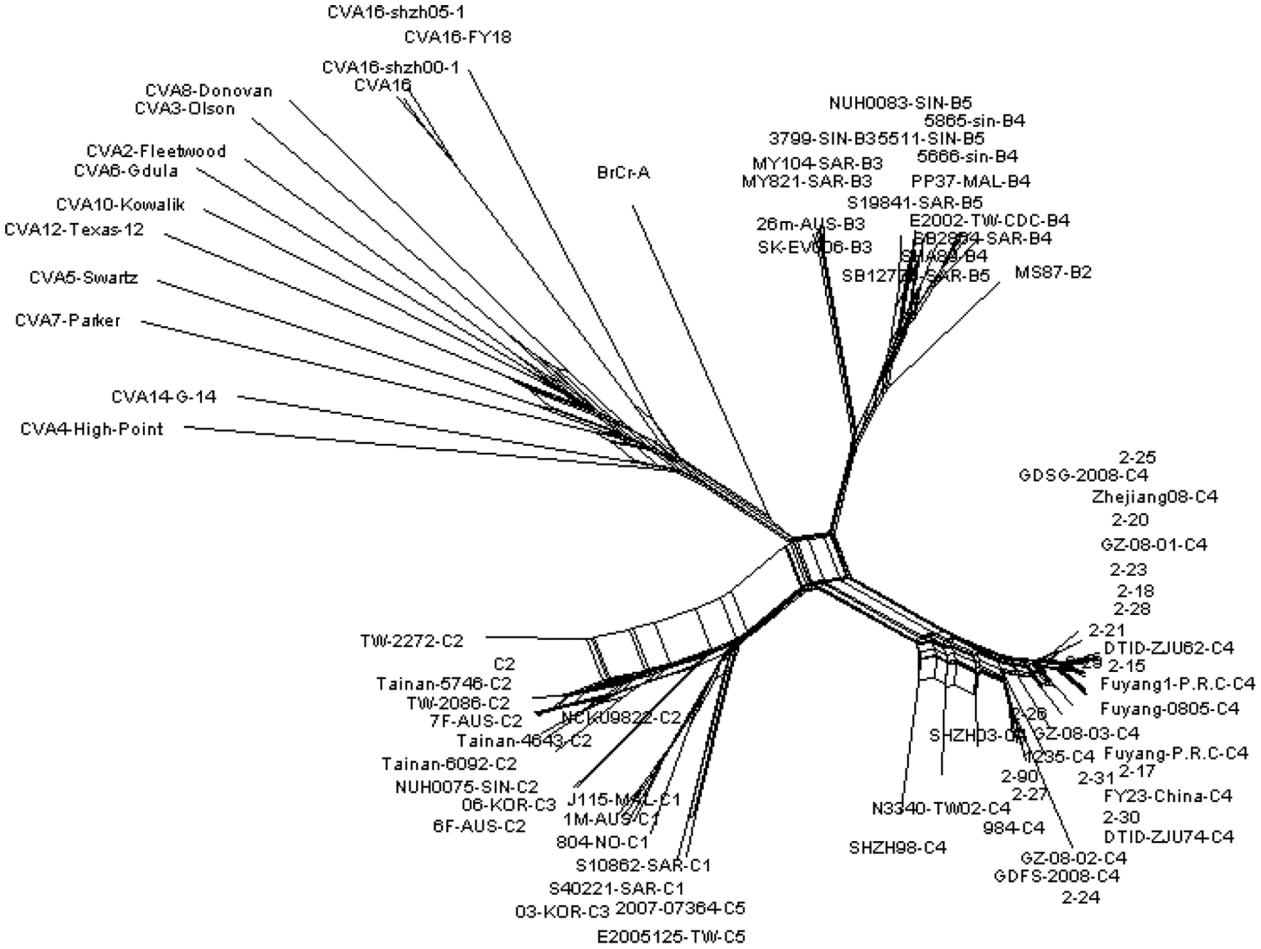
Phylogenetic tree analysis of different Taiwanese EV71 isolates using split tree method with nucleotide sequences from VP1-2A-2B region (2281 nucleotides in length). Bootstrap analysis was performed using 1000 times.

In total, 101 EV71 isolates collected were selected for the detection of new recombinant strains using the nucleotide sequences spanning the VP1-2A-2B region of EV71 genome. The results showed that all the subtypes of all the EV71 isolates can be determined clearly using Neighbor-Joining tree program (Figure 6). However, we did not find any new recombinant strain using the split tree program (Figure 7).

## Discussion

In this study, we selected 174 enterovirus 71 isolates collected at the Enterovirus Reference Lab from 2002 to 2012 to conduct a molecular epidemiological study. Although most of the specimens came from the central region of Taiwan (Figure 1), the epidemiological curve of our study (Figure 2) is very similar to the national surveillance data [15]. In addition, based on the phylogenetic tree analysis, we found that the major subtypes of EV71 infection in the central region of Taiwan are identical with those subtypes reported by other groups from either northern or southern region of Taiwan from 2002-2009 [11], [12], [16], [17]. Therefore, we believe that our findings can be extrapolated to the situation in the whole nation. Since there is no molecular epidemiological study on the EV71 subtypes in Taiwan in 2009–2012, our results may provide novel and important information for the prevention and control of this illness in Taiwan and in Asia.

It is interesting to note that, besides major strains, we found that there were minor strains of EV71 infection in almost every epidemic in Taiwan from 2002–2012 except the 2010 epidemic (Figure 2). Furthermore, the minor strains are coincident with the major strains of the subsequent outbreaks which happened several years later. For examples, we found a minor strain C4 in 2002 (1/8) and there was a major outbreak of C4 in 2005; then, in 2005, we found a minor strain B5 (1/30) and subsequently, there was a major outbreak of B5 in 2008. Similar phenomenon repeated again in 2008 when we found there is a minor strain C4 (1/51), and then there was a major outbreak of C4 in 2010 (Figure 2). Therefore, we postulated that in Taiwan, some EV71 epidemics may originate from those minor strains occurred in the previous epidemics. We further conduct phylogenetic tree analyses to show that the minor strain C4 (CVGH0007-2002) from 2002 clustered with those C4 strains found in 2004–2005 (Figure 4). Even after we included 8 EV71 C4 strains isolated from different countries in Asia from 1997 to 2004 in the analysis and we did not find any of them clustered with the Taiwanese EV71 isolates in 2004–2005 (Figure 4). Therefore, the EV71 C4 epidemic in 2004–2005 epidemic may originate from a local stain that has existed 2–3 years ago. The results indicate that EV71 minor strains may remain in the population at very low levels and the mechanism causing their outbreak deserved further analysis.

Besides originating from local strains that have existed for several years, our analyses demonstrated that some EV71 infections in Taiwan originated from its neighboring countries. In addition, the origin has been shifted from south eastern Asia countries (Malaysia and Singapore for the 2002 outbreak) to mainland China for the outbreaks recently. For examples, starting from 2010, we observed that some Taiwanese EV71 strains clustered with isolates from mainland China: the 2010 Taiwanese C4 strains clustered with EV71 strains isolated from Shanghai City and Guangdong Province, mainland China in 2009 and 2010, respectively (Figure 4); the 2011–2012 B5 strains clustered with one strain isolated from Xiamen City, Fu-Chien Province, mainland China in 2009 (Figure 5); the 2011–2012 C4 strains clustered with an EV71 strain isolated in 2009 in Henan Province, mainland China. This may due to rapid increase of cross-strait business and transportations between Taiwan and mainland China in the past few years.

In this study, we developed a RT-PCR method for the detection of new recombinant strains. Three primer sets were designed to amplify the VP1, VP2A, VP2B and partial 2C regions of EV71. The total length of the nucleotide sequence is about 2,400 bp and the genotype can be determined using both phylogenic tree and simplot analyses. Furthermore, we designed two primer sets for the amplification of the 5′UTR and 3D regions in order to identify the recombination point if a recombinant strain is identified. To fulfill this purpose, we selected 101 isolates from 2002–2012 and conduct the analysis. Unfortunately, we did not discover any new recombinant strain (Figure 7). Besides, three C4 isolates- CVGH-0002-2002, CVGH-0011-2004 and CVGH-0169-2010, were selected for bootscan analysis and their break points were located at the nucleotide position 3585 (data not shown). To further confirm our findings and to find out if there is any new recombination event that we missed in the analysis, we cut the whole region into two parts at the nucleotide position 3585 and conduct phylogenetic analysis again. The results showed that Taiwanese C4 strains clustered with other genotype C strains when their nucleotide sequences 2445–3585 spanning the VP1-2A region were analyzed. In contrast, if we used nucleotide sequences spanning 2A–2C region (nucleotide sequences 3585–4558) for the analysis, all the Taiwanese C4 strains clustered with genotype B strains (data not shown). Previously, some groups suggested that C4 should be named as a new genotype D [18], [19]. To avoid confusion, we propose to use the nomenclature system of HIV-1 [20] and designate C4 subtype as EV71 CRF01_BC.

In terms of clinical epidemiological study, we compared the clinical diagnosis, symptoms and signs between patients infected with subtypes B5 and C4 and found that patients with subtype B5 had significantly high rate of HFMD diagnosis than that of patients with subtype C4 infection (77.4% vs. 59.7%, *p*<0.05). In addition, patients with subtype C4 had significant higher rates of the following symptoms including pharyngeal vesicles or ulcers, rhinorrhea and cough (Table 1). This is the first study demonstrating that different EV71 subtypes may have different clinical manifestations. Therefore, pediatricians should be aware that high percentage of patients infected with subtype C4 may have atypical HFMD symptoms. Further study is needed to understand the correlation of sequence variations and pathogenesis between different EV71 subtypes.

In this study, we showed that from 2010 to 2012, the dominant subtypes of EV71 were C4 and B5. Since the EV71 vaccine candidate now under development by National Institute of Infectious Disease and Vaccinology of Taiwan is B4 strain, further investigation is needed to verify whether the vaccinees' immune response can cross neutralize these recent EV71 C4 and B5 strains.

## Supporting Information

Information S1 **The locations and sequences of different sets of primers used for the PCR amplification of EV71.**
(TIF)

Information S2 **Phylogenetic tree analysis of different Taiwanese and Asian EV71 isolates using Maximum likelihood method with nucleotide sequences from VP1 region (890 nucleotides in length, the whole tree).** Bootstrap analysis was performed using 1000 times. The VP1 nucleotide sequences from two CAV16 strains were used as an out group for the analysis.
(TIF)
