# Supplementary figures and images for: Molecular Epidemiology of Enterovirus 71 Infection in the Central Region of Taiwan from 2002 to 2012
Source: PLoS One. 2013 Dec 31;8(12):e83711. doi: 10.1371/journal.pone.0083711 (PMC3877097; doi:10.1371/journal.pone.0083711)

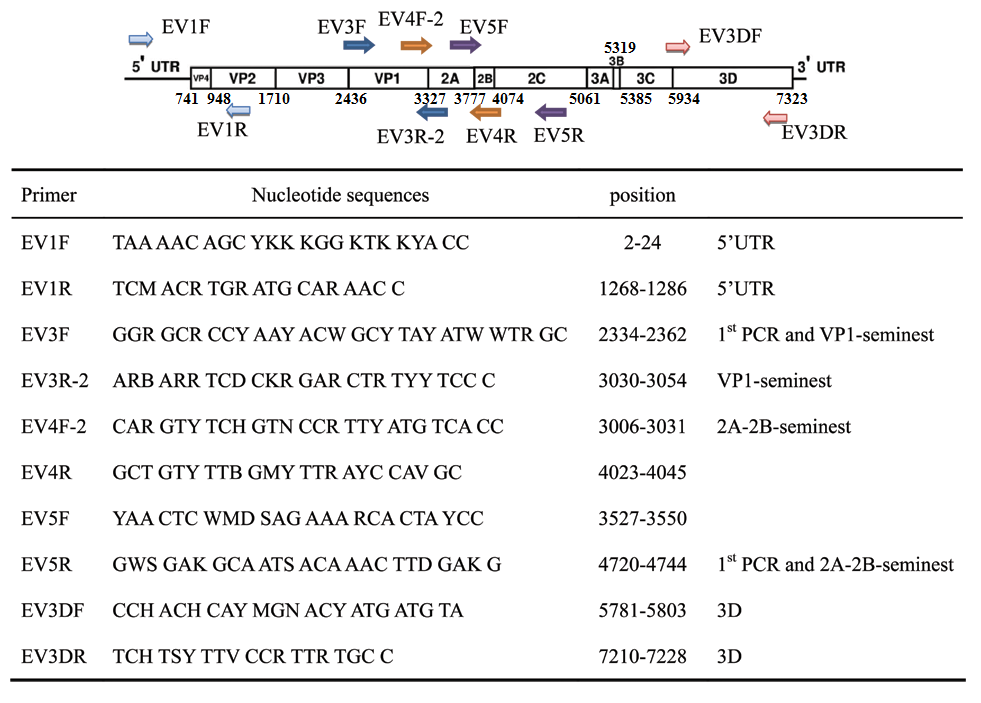

Supplement: Information S1 — The locations and sequences of different sets of primers used for the PCR amplification of EV71. (TIF) [file pone.0083711.s001.tif]

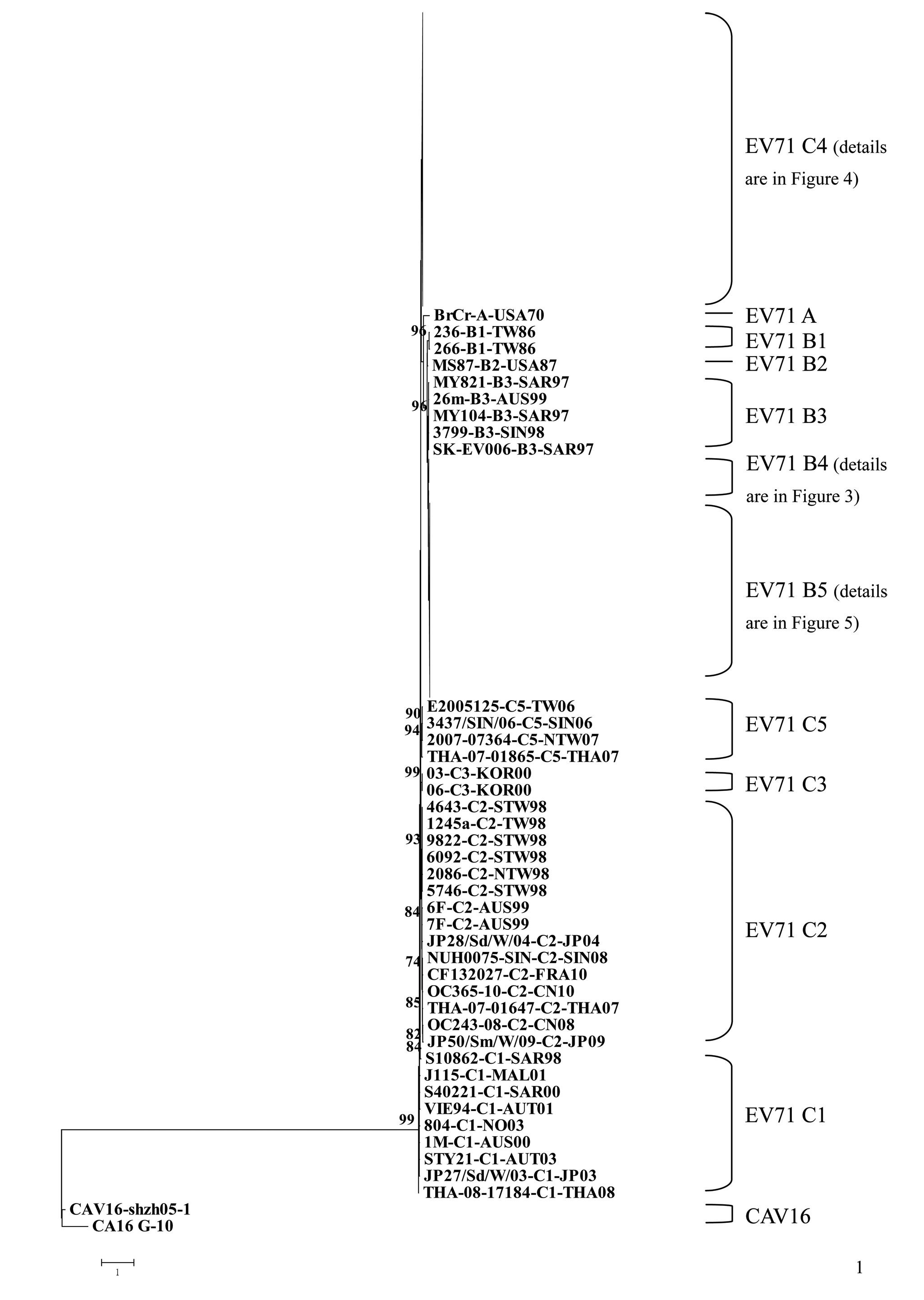

Supplement: Information S2 — Phylogenetic tree analysis of different Taiwanese and Asian EV71 isolates using Maximum likelihood method with nucleotide sequences from VP1 region (890 nucleotides in length, the whole tree). Bootstrap analysis was performed using 1000 times. The VP1 nucleotide sequences from two CAV16 strains were used as an out group for the analysis. (TIF) [file pone.0083711.s002.tif]
